# Intubation Success through I-Gel® and Intubating Laryngeal Mask Airway® Using Flexible Silicone Tubes: A Randomised Noninferiority Trial

**DOI:** 10.1155/2016/7318595

**Published:** 2016-07-10

**Authors:** Latha Naik, Neerja Bhardwaj, Indu Mohini Sen, Rakesh V. Sondekoppam

**Affiliations:** ^1^Department of Anaesthesia and Intensive Care Medicine, Postgraduate Institute of Medical Education and Research, Chandigarh 160012, India; ^2^Department of Anesthesia and Perioperative Medicine, Schulich School of Medicine and Dentistry, Western University, London, ON, Canada N6A 5A5

## Abstract

*Introduction*. The study aims to test whether flexible silicone tubes (FST) improve performance and provide similar intubation success through I-Gel as compared to ILMA. Our trial is registered in CTRI and the registration number is “CTRI/2016/06/006997.”* Methods*. One hundred and twenty ASA status I-II patients scheduled for elective surgical procedures needing tracheal intubation were randomised to endotracheal intubation using FST through either I-Gel or ILMA. In the ILMA group (*n* = 60), intubation was attempted through ILMA using FST and, in the I-Gel group (*n* = 60), FST was inserted through I-Gel airway.* Results*. Successful intubation was achieved in 36.67% (95% CI 24.48%–48.86%) on first attempt through I-Gel (*n* = 22/60) compared to 68.33% (95% CI 56.56%–80.1%) in ILMA (*n* = 41/60) (*p* = 0.001). The overall intubation success rate was also lower with I-Gel group [58.3% (95% CI 45.82%–70.78%); *n* = 35] compared to ILMA [90% (95% CI 82.41%–97.59%); *n* = 54] (*p* < 0.001). The number of attempts, ease of intubation, and time to intubation were longer with I-Gel compared to ILMA. There were no differences in the other secondary outcomes.* Conclusion*. The first pass success rate and overall success of FST through an I-Gel airway were inferior to those of ILMA.

## 1. Introduction

Supraglottic airway (SGA) devices are commonly used adjuncts to secure the airway during anesthesia or resuscitation and are an integral part of difficult airway algorithm for either elective or rescue use [1, 2]. Commonly used SGAs such as classic or proseal LMA are not ideal intubation aids as the airway conduit is either too narrow to accommodate an adult diameter endotracheal tube (ETT) or too long for the tracheal tube to reach the trachea. Additionally, CLMA is not sufficiently rigid to align the LMA with the glottic inlet [3]. The Intubating Laryngeal Mask Airway (ILMA) was introduced in 1997 for clinical use and is currently the “gold standard” for tracheal intubation through SGA either blindly or by fiberscope guidance [4–7]. I-Gel is a SGA with a noninflatable cuff made of medical grade thermoplastic called Styrene Ethylene Butadiene Styrene (SEBS). The device is known to be easier to insert [8, 9], by segregating the laryngeal opening from the oropharyngeal orifice allowing better support of the perilaryngeal structures. It results in higher sealing pressures by matching the peripharyngeal anatomy despite the absence of an inflatable cuff [10]. In a cadaveric study full glottis view was obtained in 60% of the cases soon after I-Gel insertion while some glottic opening was visible in 95% of the cases [11]. The airway channel is situated deep inside the bowl of the cuff and along with the epiglottic rest is known to ensure fresh gas flow irrespective of the downfolding of epiglottis. I-Gel has also been used as a conduit for endotracheal intubation in both mannequin and humans [12, 13].

Intubation through an ILMA requires the use of a wire-reinforced flexible silicone tube (FST). The FST is proposed to have unique features best suited for its use through an SGA and include the soft moulded tip, the position of the inflation balloon within the tube wall, and the low volume cuff all of which make it easier to enter the laryngeal inlet compared to conventional PVC tubes. The wire-reinforced FST is also known to emerge at a lower angle than a normal PVC tube (47°) and hence does not abut anteriorly against the larynx, cricothyroid membrane, or trachea. The reported first pass success rate of intubation with an ILMA using the FST is in the range of 50–78.9% [14–17] whereas the same using a PVC tube ranges within 78.5%–86.7% [18, 19]. Although FST has been used for intubation through ILMA, success rate of intubation using FST through I-Gel has not been studied in normal airways. Since FST follows the curvature of the SGA, and I-Gel has been shown to conform well with the laryngeal inlet, we hypothesised that intubation success would be noninferior between the two devices utilizing FST for intubation. The primary outcome measure was the first pass success rate and the secondary outcome measures were the overall success rate, adverse events, and time to insertion and intubation of the two devices.

## 2. Materials and Methods

**Figure 1:**
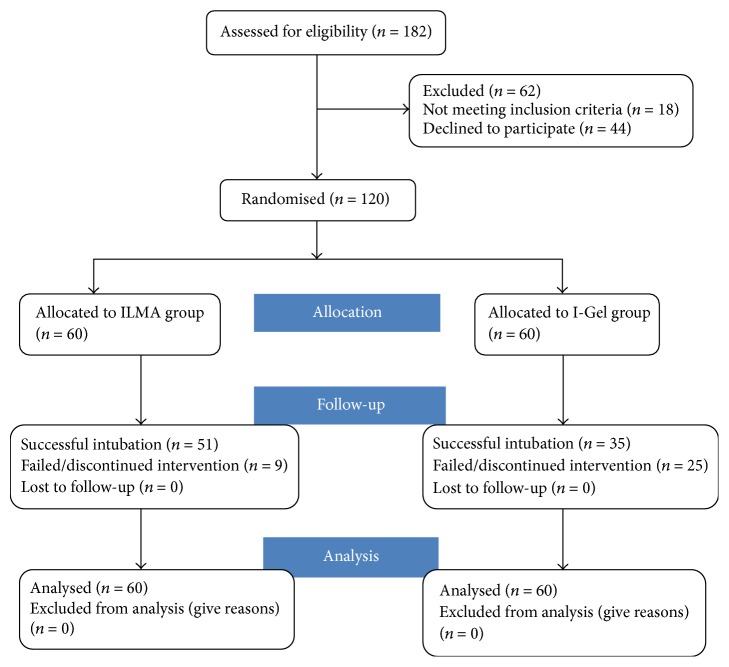
CONSORT diagram.

The study was conducted in a public tertiary care hospital in India between January 2012 and October 2012. Ethical approval for the study (8561/PG-2Trg/2010/9) was provided by the Postgraduate Institute of Medical Education and Research ethics committee, PGIMER, Chandigarh, India, on 11 December 2011. After taking informed consent, a total of 120 patients of either sex were included in the study on an intention to treat analysis basis (Figure 1: CONSORT diagram). The inclusion criteria for the study were age between 18 and 60 years, American Society of Anesthesiologists (ASA) physical status scores of I-II, 50–90 kg weight, and being scheduled to undergo elective surgical procedures requiring tracheal intubation. The patients were randomised into two groups: ILMA group (Group I, *n* = 60) and I-Gel group (Group G, *n* = 60) based on computer generated random number table (generated by NB). Patients with an ASA score III or IV, any contraindication to the use of muscle relaxants, presence of predictors of difficulty in intubation or ventilation, any increased risk of aspiration, or having a history of symptomatic gastroesophageal reflux were excluded from the study. Patients with high arched palate, restricted neck movement, or tonsillar hypertrophy were also excluded from the study.

All patients fasted overnight and received premedication with oral alprazolam 0.25 mg and ranitidine 150 mg the night before and on the morning of surgery. Preinduction monitoring included electrocardiography (ECG), noninvasive blood pressure (NIBP), and oxygen saturation (SpO_2_). Neuromuscular monitoring using train of four (TOF) was instituted after induction of anesthesia. After securing intravenous access, patients were preoxygenated for 3 minutes. Anesthesia was induced with 2 *μ*g/kg fentanyl and propofol 2 mg/kg. After confirming adequate bag-mask ventilation, atracurium 0.5 mg/kg was administered. Anesthesia was maintained with propofol infusion (100–150 *μ*g/kg/min) and 100% oxygen. After complete neuromuscular blockade (TOF count 0) the supraglottic device size 4 was inserted according to the group allocation. SGA was inserted keeping the patient's head in neutral position in both the groups and in the ILMA group the cuff was inflated with 30 mL of air. Blind intubation was attempted using 7.0 mm ID FST. Appropriate placement of the device and intubation was confirmed by observation of adequate chest expansion and appearance of ETCO_2_ waveform. Once the successful intubation was confirmed, SGA was removed using a stabilising rod. A maximum of three attempts were allowed per patient before considering the device as a failure. Intraoperatively, haemodynamic parameters were monitored every 1 minute for the first ten minutes and at 10-minute intervals thereafter till 30 minutes. Time (seconds) to successful insertion of the device (from picking the device to visible chest rise), number of attempts taken to insert the device, time to successful intubation (from the time of picking the tube from the table to visible chest rise), ease of intubation (easy/no resistance = 1, minimal resistance = 2, significant resistance = 3, or impossible = 4), airway reaction (laryngospasm, bronchospasm, coughing, and gagging), visible blood on the airway device, and any evidence of regurgitation were also noted.

On completion of the surgical procedure, propofol infusion was stopped. The duration of the surgical procedure and total propofol administered in the first hour were noted. When the TOF count was 3 or 4, neuromuscular blockade was reversed with neostigmine 0.05 mg/kg and glycopyrrolate 0.01 mg/kg. ETT was removed at TOF ratio of 90% and patient's responsiveness was assessed.

In the Post-Anaesthesia Care Unit (PACU), patients were queried for sore throat (visual analogue scale (VAS); VAS > 3 was considered significant), dysphagia (dysphagia scoring system; 0 = able to eat normal diet/no dysphagia, 1 = able to swallow some solid foods, 2 = able to swallow only semisolid foods, 3 = able to swallow liquids only, and 4 = unable to swallow anything/total dysphagia), ear/jaw/neck pain, and hoarseness of voice soon after the procedure (0 hour) and after 24 hours.

## 3. Statistical Analysis

The success rate of blind endotracheal intubation through the ILMA in first attempt using PVC has been found to be 66% [3, 4] (ranging from 50 to 80% [20]). Assuming similar success rates (74%) [18] with the use of silicone tubes in both SGA and a noninferiority limit of 30%, a total of 54 patients (27 patients per group) were required to be 80% sure that the upper limit of a one-sided 95% confidence interval (or equivalently a 90% two-sided confidence interval) will exclude a difference in favour of the standard group of more than 30% [21]. Hence we recruited a total of 120 patients (60 patients per group) to account for possible attrition of cases.

The statistical analysis was carried out using statistical package for social sciences (SPSS Inc., Chicago, IL, version 17 for windows). Normality of distribution was calculated using Shapiro-Wilk's test. Normally distributed data were compared using Student's “*t*” test and skewed data or scores were compared using Mann-Whitney *U* test. Qualitative or categorical variables were described as frequencies or proportions and compared using Fishers exact test. Repeated measures ANOVA was used for the continuous variables, namely, heart rate and systolic and diastolic blood pressure. Each variable was first tested for the assumption of sphericity using Mauchly's test. All the variables had violation of sphericity and hence the differences in the observations of a variable over time were assessed using Greenhouse-Geisser and Huynh-Feldt corrections both within groups and between the groups. If there was a significant difference within a variable, pairwise comparison was used to find out at what time intervals the individual variations differed significantly with the baseline measurement. Bootstrapping for insertion parameters of the SGA and ETT was performed with 5000 bootstrap samples and the mean (95% CI) was obtained. The bootstrapped means were also compared between the two groups. All statistical tests were 2-sided and performed at a significance level of *α* = 0.05.

## 4. Results

In this prospective, randomised clinical trial, a total of 182 patients were approached for the study (from November 2011 to October 2012) out of which 62 were found to be ineligible for the study (44 had some predictors of difficult airway; 18 did not consent for the study) and finally 120 patients were included. All recruited patients completed the study and hence were included for the final analysis (Figure 1). The two groups were comparable for age, weight, gender distribution, ASA physical status, and airway assessment (Table 1).

The number of attempts required for placement of I-Gel was comparable to that with ILMA (*p* = 0.171). The placement of I-Gel was easy in 42 patients, moderate in 16 patients, and difficult in 2 patients whereas the placement of ILMA was easy in 43 patients, moderate in 17 patients, and not difficult in any patient (*p* = 0.755). The time required for placement of I-Gel airway was comparable to that of ILMA (*p* = 0.860).

The overall intubation success rate was 58.3% (95% CI 45.82% to 70.78%) with I-Gel group (*n* = 35/60) compared to 90% (95% CI 82.41% to 97.59%) with ILMA (*n* = 54) (*p* < 0.001). The first pass success rate was 36.67% (95% CI 24.48% to 48.86%) with I-Gel (*n* = 22/60) compared to 68.33% (95% CI 56.56% to 80.1%) with ILMA (*n* = 41/60) (*p* = 0.001). The comparisons of 95% confidence intervals for the primary outcome of first pass success rate revealed the inferiority of intubating FST through the I-Gel in comparison to ILMA. The number of intubation attempts (*p* = 0.027) and time to intubation (*p* = 0.007) were significantly more with I-Gel compared to ILMA. Out of 35 successful attempts at insertion of device, intubation through I-Gel airway was easy in 17 cases, moderate in 13 cases, and difficult in 5 cases. Out of 54 successful attempts at device insertion through ILMA, intubation was easy for 39 cases, moderate for 13 cases, and difficult for 2 cases and this difference was statistically significant (*p* = 0.017) (Table 2). The bootstrapped confidence intervals for insertion parameters of SGA and ETT continued to show a statistically significant difference in time to intubation and the ease of intubation between the two groups while the rest of these parameters were comparable between the two groups (Table 3).

**Figure 2:**
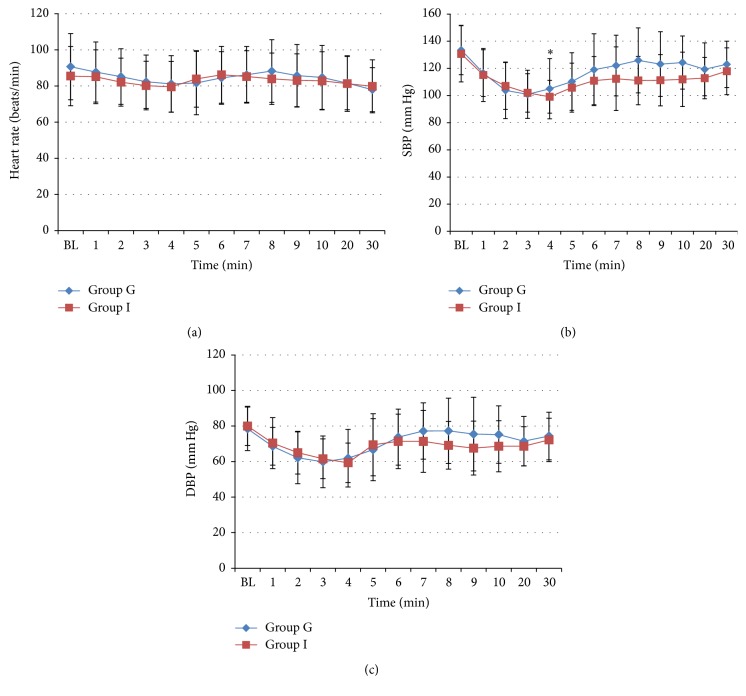
(a) Heart rate variation between two groups. (b) Systolic blood pressure variation between two groups. ^*∗*^ *p* = 0.04, repeated measures ANOVA test. (c) Diastolic blood pressure variation between two groups.

No airway reactions like gagging, laryngospasm, bronchospasm, or obstruction were noted during insertion of airway device in both groups. On removal of the airway device, laryngospasm was noted in one patient in each group. Blood on device was noted in 10 cases in I-Gel group and in 9 cases in ILMA group although this difference was not statistically significant. No patient had any evidence of gagging, bronchospasm, nausea/vomiting, or regurgitation on removal of devices in both the groups. There was no significant difference in the incidence of sore throat, dysphagia, and ear/jaw/neck pain between the two groups at 0 and 24 hours after surgery. The systolic blood pressure was lower from 1st min to 4th min after induction when compared to the baseline, but this was statistically significant only at 4th min (*p* = 0.04). At rest of the time intervals systolic blood pressure was comparable to the baseline (Figure 2). The heart rate and diastolic blood pressure did not show any significant difference compared to baseline in both I-Gel and ILMA groups. When the two groups were compared there were no significant differences in HR, SBP, and DBP. There was no significant difference in oxygen saturation both within group and in between groups. In no patient oxygen saturation (SpO_2_) decreased below 95%. EtCO_2_ levels were maintained in the normal range (35–45) in all the patients in both the groups.

## 5. Discussion

Our study shows the first pass success rate and the overall success of intubation using FST through an I-Gel airway to be inferior to that of ILMA. Intubation using FST through I-Gel had significantly higher insertion attempts and more failures and took longer intubation times in comparison to ILMA. The ease of intubation and the time to intubation were significantly longer with I-Gel even when corrected for skewed distribution using bootstrapping.

SGA is an integral part of difficult airway algorithm and resuscitation protocols [22]. It is also commonly used as a rescue device when a “cannot intubate, cannot ventilate” scenario arises [23]. In many scenarios the SGA could be used as a conduit for endotracheal intubation, either using a fiberscope or by blind passage. SGAs are also commonly used in out-of-hospital cardiopulmonary arrest (OHCA) and have been shown to reduce the time from collapse to securing the airway [24]. Recently the resuscitation outcomes such as survival to hospital discharge, return of spontaneous circulation, and 24-hour survival were shown to be better following endotracheal intubation in comparison to SGA when used for OHCA [25, 26]. Intubating through a SGA may thus potentially combine the best of both the devices for such scenarios. Among the studies comparing intubation success through ILMA versus I-Gel, most of the studies have utilized PVC endotracheal tubes through the I-Gel while using FST or PVC tubes through the ILMA [18, 19, 27]. Halwagi et al. [18] used PVC ETT through both devices and found that first pass success rate is 69% with the I-Gel and 74% with ILMA. The overall success rate was 73% with I-Gel and 91% with ILMA. A similar study by Kapoor et al. [19] showed that the first pass success rate of PVC ETT through the two devices was 66 and 74% while the overall success rate of intubation was 96 and 82% in I-Gel and ILMA, respectively. In a study comparing different endotracheal tubes (FST versus PVC tubes) through I-Gel and single use ILMA in patients with difficult airways, Theiler et al. [27] noted a blind intubation success rate of 15% with McGill PVC tubes and 21% with single use FST through I-Gel while the same was 69 and 60% with ILMA, respectively. The patients in the study by Theiler et al. [27] had some predictors of difficult airway which may explain the lower intubation success in their study in comparison to the present study.

The success rate of FST passage through ILMA in our study is in agreement with other studies. Whether FST is the ideal tube for blind endotracheal intubation through SGA or whether ordinary PVC tubes can be used instead has been studied by many investigators. Sharma et al. [28] found the first pass success rate through ILMA for FST to be similar to that of PVC tubes (95 versus 90 for FST and PVC, resp.). The overall success rate was 97 and 96% for FST and PVC tubes, respectively, but the time taken to perform the intubation and the number of manoeuvres were higher for PVC tubes. A recent study by Shah et al. [29] has also shown similar success rates between FST and PVC tubes through ILMA. While a study by Kundra et al. [30] showed a similar first pass (86%) and overall success rate of intubation (96%) for FST and PVC tubes, Kanazi et al. [31] showed an inferior performance of both Parker tube and PVC tubes through ILMA. The prewarming of PVC tubes [30] and use of repositioning manoeuvres did influence the success rates in both the studies [30, 31]. Although comparative studies of different endotracheal tubes through I-Gel are limited to a few studies, studies looking at the first pass and overall intubation success of PVC tubes through I-Gel report range within 65–69% and 73–87.7%, respectively [32, 33].

**Figure 3:**
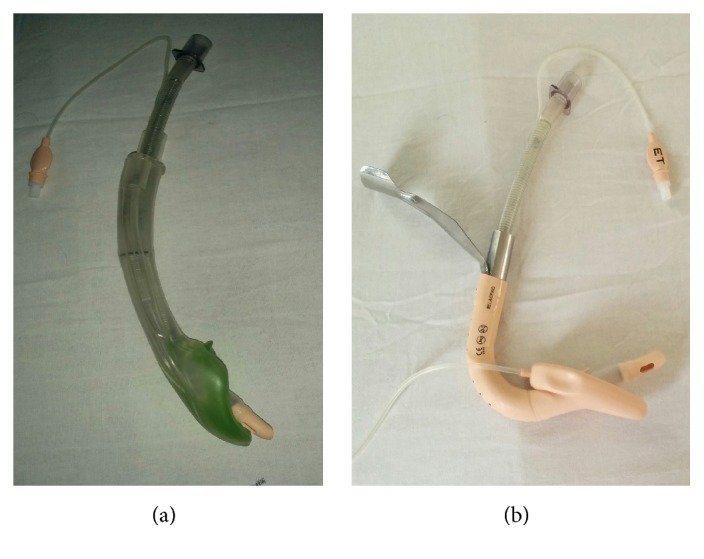


The overall success rate was significantly lower with I-Gel in our study (58.3%) and also intubation times were significantly longer in I-Gel group than in the ILMA group. The study by Halwagi et al. [18] contrastingly showed a longer intubation times with ILMA in comparison to I-Gel and could reflect the improvement in intubation success with ILMA on subsequent attempts in their study. The higher first pass and overall failure rate seen with I-Gel in comparison to ILMA could be explained based on the structural properties of the SGA and the FST. The flexible silicon tipped tube is a well-designed, straight, soft, wire-reinforced silicon tube which lacks wire reinforcement in the distal inch and terminates like a conical soft tip for use with ILMA. This combined with the enhanced curved shape of the ILMA leads these flexible tubes towards the plane of the glottis at an angle of 35°. The relatively straight shape of the I-Gel stem and the ending of the airway channel deep into the bowl of the cuff may direct the soft tip of FST posteriorly thereby increasing the risk of oesophageal intubation or snaring on the arytenoids (Figure 3). The more rigid PVC tubes have a fixed curvature directed anteriorly thereby better aligning the tube towards laryngeal inlet than FST when advanced through an I-Gel.

There are several limitations in our study. The patient population included in this study consists of patients with normal airways. Our current findings might not apply to patients with difficult airways. We did not perform any external laryngeal manipulation or use cricoid pressure in our study which could also have impacted the success rate of intubation in either group [33]. Although the intubations were performed by a single experienced performer, blinding to the device is not possible which may be a source of bias. Lack of visualisation of the position of the devices in relation to the laryngeal structures is another possible limitation of the present study. In addition a good mask larynx relationship was ascertained clinically rather than from fibreoptic view.

In conclusion, the first pass success and overall success rate of FST through I-Gel are inferior to those through ILMA. Further studies incorporating bronchoscopic visualisation for intubation attempts and manoeuvres to reposition the airway device may better illustrate the reason for intubation failure in I-Gel airway when FST are used.

## Figures and Tables

**Table 1:** Patient characteristics.

Variable	Group G (*n* = 60) Median (95% CI)	Group I (*n* = 60) Median (95% CI)	*p* value (sig < 0.05)
Age	32.00 (28.52, 42.82)	42.00 (35.68; 46.52)	0.94
Weight	60.00 (58.88; 64.86)	58.00 (56.52; 63.98)	0.75
Sex (M : F)	13 : 47	17 : 43	0.39
Duration of surgery	75.00 (68.74; 115.26)	77.50 (71.34; 95.16)	0.86
ASA status (1 : 2)	50 : 10	43 : 17	0.12
MMP (1 : 2)	35 : 25	33 : 27	0.71
TMD (cm)	6.55 (6.55; 6.68)	6.50 (6.53; 6.66)	0.57

ASA: American Society of Anesthesiologists physical status; MMP: Mallampati score; TMD: thyromental distance.

**Table 2:** Device insertion and intubation parameters.

Variable	Group G (*n* = 60) Median (95% CI)	Group I (*n* = 60) Median (95% CI)	*p* value (sig < 0.05)
Number of SGA attempts	1.00 (1.19; 2.01)	1.00 (1.22; 1.98)	0.171
Ease of SGA insertion	1.00 (1.05; 1.75)	1.00 (1.01; 1.39)	0.755
Time to SGA placement	9.28 (7.99; 13.25)	8.50 (7.54; 10.86)	0.860
Ease of intubation	2.00 (1.41; 1.91)	1.00 (1.17; 1.46)	0.017^*∗*^
Intubation success	58.3% (*n* = 35)	90% (*n* = 54)	0.000^*∗*^
Time to intubation	16.10 (13.49; 18.84)	7.90 (7.81; 13.87)	0.007^*∗*^
Number of intubation attempts	1.00 (0.94; 1.33)	1.00 (0.95; 1.15)	0.027^*∗*^
Attempts at intubation; number (percentage)			
One	22 (36.67)	41 (68.3)	0.001^*∗*^
Two	8 (13.33)	9 (15.0)	
Three	5 (8.3)	4 (6.67)	
VAS 0	1.00 (0.36; 1.37)	1.00 (1.04; 2.16)	0.833
Dysphagia 0	1.00 (0.92; 1.21)	1.00 (1.04; 1.46)	0.853
VAS 24	0.00 (−0.03; 0.43)	0.50 (0.28; 0.92)	0.052
Dysphagia 24	0.00 (−0.03; 0.15)	0.00 (−0.02; 0.06)	0.299

SGA: supraglottic airway device; VAS: visual analogue scale; *∗* indicates *p* < 0.05.

**Table 3:** Bootstrapped mean (95% CI) for device insertion and intubation parameters.

Variable	Group G (*n* = 60) BS mean (95% CI)	Group I (*n* = 60) BS mean (95% CI)	*p* value (sig < 0.05)
Number of SGA attempts	1.20 (1.07; 1.36)	1.04 (1.00; 1.08)	0.088
Ease of insertion	1.40 (1.22; 1.60)	1.24 (1.14; 1.35)	0.180
Ease of intubation	1.66 (1.43; 1.89)	1.31 (1.19; 1.46)	0.023^*∗*^
Time to SGA placement (min)	10.27 (9.07; 11.65)	9.60 (8.80; 10.44)	0.418
Time to intubation (min)	13.39 (11.41; 15.39)	10.11 (8.76; 11.53)	0.012^*∗*^
Number of intubation attempts	1.51 (1.29; 1.76)	1.31 (1.17; 1.48)	0.191

SGA: supraglottic airway device; *∗* indicates *p* < 0.05.
